# Possible role of *EMID2 *on nasal polyps pathogenesis in Korean asthma patients

**DOI:** 10.1186/1471-2350-13-2

**Published:** 2012-01-04

**Authors:** Charisse Flerida Arnejo Pasaje, Joon Seol Bae, Byung-Lae Park, Hyun Sub Cheong, Jeong-Hyun Kim, An-Soo Jang, Soo-Taek Uh, Choon-Sik Park, Hyoung Doo Shin

**Affiliations:** 1 Department of Life Science, Sogang University, Seoul, 121-742, Republic of Korea; 2 Department of Genetic Epidemiology, SNP Genetics, Inc., Seoul, 153-803, Republic of Korea; 3 Division of Allergy and Respiratory Medicine, Soonchunhyang University Seoul Hospital, Seoul, 140-743, Republic of Korea; 4 Genome Research Center for Allergy and Respiratory Diseases, Division of Allergy and Respiratory Medicine, Soonchunhyang University Bucheon Hospital, Bucheon, 420-767, Republic of Korea

## Abstract

**Background:**

Since subepithelial fibrosis and protruded extracellular matrix are among the histological characteristics of polyps, the *emilin/multimerin domain-containing protein 2 *(*EMID2*) gene is speculated to be involved in the presence of nasal polyps in asthma and aspirin-hypersensitive patients.

**Methods:**

To investigate the association between *EMID2 *and nasal polyposis, 49 single-nucleotide polymorphisms (SNPs) were genotyped in 467 asthmatics of Korean ancestry who were stratified further into 114 aspirin exacerbated respiratory disease (AERD) and 353 aspirin-tolerant asthma (ATA) subgroups. From pairwise comparison of the genotyped polymorphisms, 14 major haplotypes (frequency > 0.05) were inferred and selected for association analysis. Differences in the frequency distribution of *EMID2 *variations between polyp-positive cases and polyp-negative controls were determined using logistic analyses.

**Results:**

Initially, 13 *EMID2 *variants were significantly associated with the presence of nasal polyps in the overall asthma group (*P *= 0.0008-0.05, OR = 0.54-1.32 using various modes of genetic inheritance). Although association signals from 12 variants disappeared after multiple testing corrections, the relationship between *EMID2_BL1_ht2 *and nasal polyposis remained significant via a codominant mechanism (*P^corr ^*= 0.03). On the other hand, the nominal associations observed between the genetic variants tested for the presence of nasal polyps in AERD (*P *= 0.003-0.05, OR = 0.25-1.82) and ATA (*P *= 0.01-0.04, OR = 0.46-10.96) subgroups disappeared after multiple comparisons, suggesting lack of associations.

**Conclusions:**

These preliminary findings suggest that *EMID2_BL1_ht2 *may be a susceptibility marker of inflammation of the nasal passages among Korean asthma patients.

## Background

Nasal polyps are abnormal lesions arising mainly from the nasal mucosa and paranasal sinuses. The histopathologic characteristics of these polyps include extensive thickening of the basement membrane due to deposition of fibronectin and collagens, an event that is referred to as subepithelial fibrosis [1]. Nasal polyposis occur more frequently in asthma and aspirin hypersensitive patients [2], resulting in symptoms of airways bronchoconstriction and mucus hypersecretion, and posing threats of respiratory failure in affected individuals. Despite previous attempts to explain disease pathogenesis, the exact genetic mechanisms underlying the development of nasal polyps in asthma patients are still unclear and would benefit from further research.

Recently, the human *emilin/multimerin domain-containing protein 2 *(*EMID2*) gene has been implicated as a potential marker of aspirin exacerbated respiratory disease (AERD), a condition that is characterized by the presence of nasal polyps in nasal passages [3]. Emilin and multimerin are glycoproteins that act as major components of the extracellular matrix (ECM) [4], a cellular structure that is accumulated in diseases of the airways. Mapped to the 7q22.1 locus and spanning over 194 kb with 13 exons, *EMID2 *codes for a protein that encodes three identical collagen α1 (XXVI) chains (COL26A1) which comprises 438 amino acids in length [5]. In addition to collagen deposition in the basement membrane as a feature of nasal polyps, subepithelial fibrosis has also been implicated in low forced expiratory volume in one second (FEV_1_), a common parameter used in assessing bronchial constriction. Furthermore, EMID2 binds to a gene [5] that has recently been shown to be highly expressed in nasal mucosa,[6] providing a link between *EMID2 *and nasal polyposis.

Despite the relevance of *EMID2 *in various cellular processes, little is known regarding the involvement of the gene in human diseases. With the accumulation of the ECM in nasal polyp tissues and the crucial role of subepithelial fibrosis in disease pathogenesis, a case-control study was conducted to investigate the association between variations in *EMID2 *and the presence of nasal polyps among Korean asthma patients.

## Methods

### Study patients

Asthma patients from Korean hospitals belonging to the Asthma Genome Research Center were recruited for the study. Written informed consent was secured from each patient before blood was drawn, and the study protocols were approved by the Institutional Review Board of each participating hospital. Following the guidelines of Global Initiative for Asthma (GINA), asthma was diagnosed as described previously [7]. Twenty-four common inhalant allergens were used in a skin-prick test (Bencard Co. Ltd., Brentford, UK), and atopy was defined as at least a 3-mm wheal reaction to any of the allergens. Furthermore, total immunoglobulin E (IgE) was measured using the CAP system (Pharmacia Diagnostics, Uppsala, Sweden). Asthmatics with endoscopically visible polyps present in the middle nasal meatus were categorized as polyp-positive cases while the rest were identified as polyp negative controls.

To distinguish AERD patients from aspirin-tolerant asthma (ATA) subgroups, all asthma patients underwent oral aspirin challenge (OAC) that was performed according to our previous methods [7]. Asthmatics exhibiting ≥ 20% decrease in FEV_1 _or a 15-19% decrease in FEV_1 _with naso-ocular or cutaneous reactions were categorized in the AERD group, whereas those demonstrating < 15% decrease in FEV_1 _without naso-ocular or cutaneous reactions classified into the aspirin-tolerant asthma (ATA) group.

### Selection and genotyping of single-nucleotide polymorphisms

Tagging single-nucleotide polymorphisms (SNPs) in the *EMID2 *gene were selected and screened from the International HapMap database (version: release #27; http://www.hapmap.org) based on linkage disequilibrium (LD) status in the Asian population (Chinese Hans and Japanese), locations (SNPs in exons were preferred) and amino acid changes (non-synonymous SNPs were preferred). From the minor allele frequency (MAF) scores, LD relations between the screened SNPs were evaluated using the Haploview software (Cambridge, MA, USA; http://www.broad.mit.edu/mpg/haploview). SNPs having MAF > 0.05 and tagging SNPs if several polymorphisms showed high LD (> 0.98) were selected for genotyping that was performed using TaqMan assay [8] in the ABI prism 7900HT sequence detection system (Applied Biosystems Foster City, CA, USA). Genotyped data quality was assessed by duplicate DNA checking (n = 10; rate of concordance in duplicates > 99%). Using the PHASE algorithm ver. 2.0 software [9], haplotypes were inferred from the successfully genotyped SNPs of the entire study population and those with frequency of over 0.05 were selected for association analyses in the overall asthma patients as well as the AERD and ATA subgroups.

### Statistical analyses

LD between all pairs of biallelic loci were determined using Lewontin's D' (|*D'*|) and LD coefficient *r^2 ^*were examined using the Haploview algorithm [10]. Differences in the genotype distributions of *EMID2 *variations in polyp positive asthma cases and polyp negative asthma controls were analyzed using logistic models adjusted for age of initial diagnosis (continuous value), sex (male = 0, female = 1), smoking status (non-smoker = 0, ex-smoker = 1, smoker = 2) and atopy (absence = 0, presence = 1) to eliminate confounding variables that might influence the findings. AERD status was also controlled for the logistic analysis of the overall asthma subjects. Data were managed on the Statistical Analysis System (SAS) version 9.1 (SAS Inc., Cary, NC). Statistical power of single associations was determined using the Power for Genetic Association Analyses (PGA) software [11], and multiple testing corrections was calculated using the effective number of independent marker loci (Meff) that accounts for the eigenvalue spectral decomposition (SpD) of all the genotypes represented in the correlation matrix [12] that was extracted from the SNPSpD program.

## Results

### Classifications and clinical characteristics of the study patients

From a total of 467 Korean asthmatics recruited for the study, 158 patients were categorized as polyp-positive cases while 309 subjects were identified as polyp negative controls. Based on individual reaction to OAC, the overall study patients were stratified further into 114 AERD patients (including 66 polyp-positive cases and 48 polyp-negative controls) and 353 ATA subjects (including 92 polyp-positive cases and 261 polyp-negative controls). Fall rate of FEV_1 _by aspirin provocation (polyp-positive = 12.22 vs. polyp-negative = 6.78) and a positive rate of aspirin intolerance (polyp-positive = 41.77 vs. polyp-negative = 15.53) were significantly different between polyp-positive cases and polyp-negative controls (*P *= 0.0001, Table 1). The demographics and clinical profiles of the study patients are summarized in Table 1.

**Table 1:** Clinical profile of asthmatic patients (n = 467)

Clinical profile	Polyp-positive	Polyp-negative	*P*-value
Number of subjects (n)	158	309	
Age [year, mean (range)]	46.24 (17.93-76.86)	47.00 (15.40 - 77.88)	0.56
Sex (n, male/female)	55/103	100/209	0.60
Total smoker (Current Smoker; Ex-Smoker) (%)	27.21 (11.39; 15.82)	27.83 (11.33; 16.50)	0.93
Body mass index (kg/m^2^)	23.95 ± 3.00	24.53 ± 3.51	0.06
% fall of FEV_1 _by aspirin provocation	12.22 ± 14.39	6.78 ± 11.46	**< 0.0001**
Blood eosinophil (%)	6.92 ± 6.23	5.98 ± 6.00	0.12
PC20 methacholine (mg/ml)	5.83 ± 8.87	6.88 ± 8.62	0.23
Total IgE (IU/ml)	298.35 ± 469.67	368.39 ± 654.01	0.19
FEV_1 _(% predicted)	89.67 ± 15.76	91.79 ± 17.33	0.18
FVC (% predicted)	89.02 ± 12.65	87.68 ± 14.56	0.30
Positive rate of skin test (%)	51.90	57.61	0.24
Positive rate of aspirin intolerance (%)	41.77	15.53	**< 0.0001**

Values are mean ± SE. BMI, body mass index.

### Distribution of *EMID2 *variants

**Figure 1:**
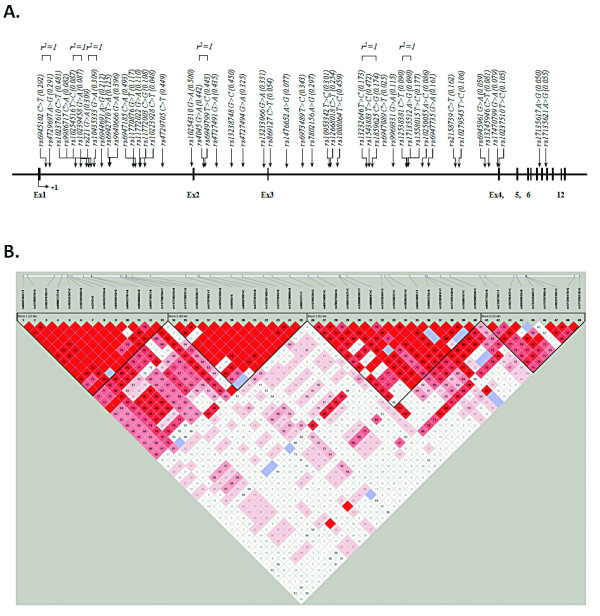
**Physical map and LD of the *EMID2 *gene**. A. Schematic gene map and SNPs of *EMID2 *on chromosome 7q22.1. Black blocks represent coding exons and white blocks represent 5' and 3' UTR. The first base of translation site was denoted as nucleotide +1. SNPs in absolute linkage are indicated by brackets (*r^2 ^*= 1). B. LD coefficient (|D'|) among *EMID2 *SNPs in a Korean population. Red blocks indicate |D'| = 1, LOD ≥ 2, blue blocks |D'| = 1, LOD < 2 and white blocks |D'| < 1, LOD < 2. UTR, untranslated region.

With an average call rate of 99.9%, 49 intronic SNPs of *EMID2 *were successfully genotyped in Korean asthma patients (Additional file 1: Table S1). Four LD blocks were inferred from pairwise comparison of all genotypes polymorphisms, and 14 major haplotypes with frequencies over 0.05 (Figure 1; Additional file 2: Figure S1) were tested for association with the presence of nasal polyps among asthma patients.

### Association analysis of *EMID2 *variants with nasal polyps in asthma patients

Results of logistic analysis for the overall asthma patients initially revealed significant associations of ten *EMID2 *SNPs (*rs6945102*, *rs4729697*, *rs221*, *rs10435333*, *rs6947185*, *rs4727494*, *rs13233066*, *rs1008064*, *rs1543883*, and *rs13245946*) with the presence of nasal polyps (*P *= 0.004-0.05, OR = 0.61-1.32 depending on the genetic model; Table 2 and Table S2). However, with a Meff of 42.7377 used to correct the *P *- values, the significant signals disappeared after multiple testing corrections. Furthermore, signals from *EMID2_BL1_ht2 *(unique to the minor alleles of *rs6945102*, *rs4729697*, *rs221*, and *rs10435333*, among the haplotypes with frequency > 0.05), *EMID2_BL2_ht2 *(unique to the minor alleles of *rs4727494 *and *rs13233066*), and *EMID2_BL3_ht1 *(unique to the minor alleles of *rs1008064 *and *rs1543883*) achieved significance (*P *= 0.0008-0.03, OR = 0.54-0.74 depending on the genetic model; Table 2 and Table S2) after analyzing the differences in the frequency distribution of *EMID2 *haplotypes between polyp-positive and polyp-negative asthma patients. Although multiple testing comparisons reduced the values from two haplotypes to nominal evidence of association, the association signal of *EMID2_BL1_ht2 *remained significant via a codominant mechanism (*P^corr ^*= 0.03; Table 2 and Table S2).

**Table 2:** Association of significant *EMID2 *variants with nasal polyps in the overall asthmatic patients (n = 467)

SNP/Haplotype	MAF		Co-dominant			Dominant			Recessive			Statistical Power
	
	Polyp-positive (n = 158)	Polyp-negative (n = 309)	OR(95%CI)	P*	P^corr^**	OR(95%CI)	P*	P^corr^**	OR(95%CI)	P*	P^corr^**	
*rs6945102C > T*	0.228	0.322	0.62(0.45-0.87)	**0.005**	NS	0.60(0.40-0.91)	**0.02**	NS	0.39(0.16-0.92)	**0.03**	NS	62.03
*rs4729697A > G*	0.224	0.321	0.61(0.44-0.86)	**0.004**	NS	0.59(0.39-0.89)	**0.01**	NS	0.39(0.16-0.93)	**0.03**	NS	60.92
*rs221G > A*	0.252	0.337	0.67(0.49-0.93)	**0.02**	NS	0.64(0.43-0.97)	**0.04**	NS	0.48(0.22-1.06)	0.07	-	66.93
*rs10435333G > A*	0.252	0.337	0.67(0.48-0.93)	**0.02**	NS	0.64(0.43-0.97)	**0.03**	NS	0.49(0.22-1.07)	0.07	-	66.93
*rs6947185C > A*	0.433	0.537	0.69(0.52-0.92)	**0.01**	NS	0.69(0.44-1.09)	0.11	-	0.53(0.32-0.87)	**0.01**	NS	72.99
*rs4727494G > A*	0.263	0.359	0.65(0.47-0.90)	**0.009**	NS	0.65(0.43-0.98)	**0.04**	NS	0.40(0.18-0.87)	**0.02**	NS	66.22
*rs13233066G > A*	0.275	0.362	0.70(0.51-0.95)	**0.02**	NS	0.65(0.43-0.97)	**0.04**	NS	0.60(0.30-1.19)	0.14	-	69.05
*rs1008064T > C*	0.494	0.430	1.32(1.00-1.75)	**0.05**	NS	1.46(0.94-2.26)	0.10	-	1.48(0.92-2.38)	0.10	-	77.44
*rs1543883T > C*	0.506	0.450	1.32(1.00-1.76)	**0.05**	NS	1.51(0.96-2.38)	0.08	-	1.41(0.88-2.25)	0.16	-	78.55
*rs13245946C > T*	0.108	0.073	1.53(0.95-2.45)	0.08	-	1.36(0.79-2.33)	0.26	-	13.01(1.45-116.41)	**0.02**	NS	41.62
*EMID2_BL1_ht2*	0.180	0.290	0.54(0.37-0.77)	**0.0008**	**0.03**	0.53(0.35-0.82)	**0.004**	NS	0.22(0.06-0.74)	**0.01**	NS	71.84
*EMID2_BL2_ht2*	0.259	0.356	0.64(0.47-0.89)	**0.007**	NS	0.63(0.42-0.95)	**0.03**	NS	0.42(0.19-0.92)	**0.03**	NS	81.64
*EMID2_BL3_ht1*	0.453	0.510	0.74(0.56-0.98)	**0.03**	NS	0.66(0.42-1.02)	0.06	-	0.67(0.42-1.08)	0.10	-	88.51

*P-values at 0.05 level of significance adjusted for initial diagnosed age, sex, smoking status, atopy and AERD. Significant values are shown in bold.

**P-values after multiple testing corrections (Meff = 42.7377).

MAF, minor allele frequency; OR, odds ratio; CI, confidence interval; NS, not significant.

In further association analysis, 17 *EMID2 *variations (*rs6945102*, *rs4729697*, *rs10237610*, *rs221*, *rs10435333*, *rs9640666*, *rs6947185*, *rs4729705*, *rs10254310*, *rs6949799*, *rs4727491*, *rs13238748*, *rs4727494*, *rs13233066*, *EMID2_BL1_ht1*, *EMID2_BL1_ht2*, and *EMID2_BL2_ht2*) were initially correlated with the presence of nasal polyps in the AERD subgroup (*P *= 0.003-0.05, OR = 0.25-1.82 depending on the genetic model; Table 3 and Table S3). However, the association signals disappeared after multiple testing corrections. Similarly, significant *P *- values of *EMID2 *variants (*rs6947185*, *rs12538381*, *rs17135512*, *rs13245946*, *EMID2_BL1_ht2*, *EMID2_BL4_ht1*) tested for polyp development in the ATA subgroup (*P *= 0.01-0.04, OR = 0.46-10.96 depending on the genetic model; Table 4 and Table S4) did not reach the threshold of multiple testing corrections.

**Table 3:** Association of significant *EMID2 *variants with nasal polyps in AERD patients (n = 114)

SNP/Haplotype	MAF		Co-dominant			Dominant			Recessive		
	
	Polyp-positive (n = 66)	Polyp-negative (n = 48)	OR(95%CI)	P*	P^corr^**	OR(95%CI)	P*	P^corr^**	OR(95%CI)	P*	P^corr^**
*rs6945102C > T*	0.205	0.365	0.43(0.22-0.84)	**0.01**	NS	0.39(0.17-0.86)	**0.02**	NS	0.27(0.05-1.51)	0.14	-
*rs4729697A > G*	0.200	0.365	0.42(0.22-0.83)	**0.01**	NS	0.38(0.17-0.84)	**0.02**	NS	0.27(0.05-1.57)	0.15	-
*rs10237610C > T*	0.554	0.438	1.82(1.01-3.29)	**0.05**	NS	1.50(0.60-3.73)	0.38	-	3.42(1.22-9.60)	**0.02**	NS
*rs221G > A*	0.208	0.396	0.35(0.18-0.70)	**0.003**	NS	0.35(0.16-0.78)	**0.01**	NS	0.10(0.01-0.87)	**0.04**	NS
*rs10435333G > A*	0.212	0.396	0.36(0.18-0.71)	**0.003**	NS	0.36(0.16-0.81)	**0.01**	NS	0.09(0.01-0.85)	**0.04**	NS
*rs9640666G > A*	0.326	0.479	0.46(0.25-0.86)	**0.02**	NS	0.35(0.15-0.84)	**0.02**	NS	0.43(0.14-1.35)	0.15	-
*rs6947185C > A*	0.400	0.531	0.52(0.28-0.94)	**0.03**	NS	0.27(0.10-0.74)	**0.01**	NS	0.65(0.25-1.69)	0.37	-
*rs4729705C > T*	0.371	0.521	0.50(0.28-0.88)	**0.02**	NS	0.25(0.10-0.63)	**0.003**	NS	0.66(0.25-1.70)	0.38	-
*rs10254310G > A*	0.432	0.552	0.58(0.34-1.01)	0.06	-	0.39(0.15-0.98)	**0.04**	NS	0.60(0.25-1.46)	0.26	-
*rs6949799T > C*	0.546	0.417	1.77(1.02-3.08)	**0.04**	NS	1.83(0.77-4.34)	0.17	-	2.61(1.00-6.80)	**0.05**	NS
*rs4727491G > A*	0.538	0.417	1.74(1.00-3.05)	**0.05**	NS	1.83(0.77-4.34)	0.17	-	2.48(0.95-6.51)	0.06	-
*rs13238748G > C*	0.371	0.521	0.50(0.28-0.88)	**0.02**	NS	0.25(0.10-0.63)	**0.003**	NS	0.66(0.25-1.70)	0.38	-
*rs4727494G > A*	0.220	0.385	0.46(0.25-0.86)	**0.01**	NS	0.37(0.16-0.84)	**0.02**	NS	0.36(0.10-1.37)	0.14	-
*rs13233066G > A*	0.227	0.385	0.49(0.27-0.90)	**0.02**	NS	0.37(0.16-0.84)	**0.02**	NS	0.45(0.13-1.58)	0.21	-
*EMID2_BL1_ht1*	0.538	0.417	1.83(1.02-3.26)	**0.04**	NS	1.38(0.58-3.31)	0.47	-	4.23(1.42-12.57)	**0.01**	NS
*EMID2_BL1_ht2*	0.159	0.323	0.37(0.18-0.77)	**0.008**	NS	0.34(0.15-0.77)	**0.01**	NS	0.22(0.02-2.18)	0.19	-
*EMID2_BL2_ht2*	0.220	0.385	0.46(0.25-0.86)	**0.01**	NS	0.37(0.16-0.84)	**0.02**	NS	0.36(0.10-1.37)	0.14	-

*P-values at 0.05 level of significance adjusted for initial diagnosed age, sex, smoking status, and atopy. Significant values are shown in bold.

**P-values after multiple testing corrections (Meff = 42.7377).

MAF, minor allele frequency; OR, odds ratio; CI, confidence interval; NS, not significant.

**Table 4:** Association of significant *EMID2 *variants with nasal polyps in ATA patients (n = 353)

SNP/Haplotype	MAF		Co-dominant			Dominant			Recessive		
	
	Polyp-positive (n = 92)	Polyp-negative (n = 261)	OR(95%CI)	P*	P^corr^**	OR(95%CI)	P*	P^corr^**	OR(95%CI)	P*	P^corr^**
*rs6947185C > A*	0.457	0.538	0.72(0.51-1.01)	0.06	-	0.86(0.50-1.50)	0.60	-	0.46(0.25-0.84)	**0.01**	NS
*rs12538381C > T*	0.109	0.094	1.18(0.69-2.05)	0.55	-	1.01(0.55-1.86)	0.99	-	10.96(1.08-111.20)	**0.04**	NS
*rs17135512A > G*	0.109	0.094	1.18(0.69-2.05)	0.55	-	1.01(0.55-1.86)	0.99	-	10.96(1.08-111.20)	**0.04**	NS
*rs13245946C > T*	0.125	0.069	1.85(1.08-3.16)	**0.02**	NS	1.69(0.91-3.14)	0.10	-	13.43(1.44-125.46)	**0.02**	NS
*EMID2_BL1_ht2*	0.196	0.284	0.61(0.41-0.93)	**0.02**	NS	0.65(0.39-1.06)	0.08	-	0.20(0.05-0.88)	**0.03**	NS
*EMID2_BL4_ht1*	0.266	0.201	1.42(0.96-2.09)	0.08	-	2.68(1.05-6.85)	**0.04**	NS	1.34(0.83-2.18)	0.24	-

*P-values at 0.05 level of significance adjusted for initial diagnosed age, sex, smoking status, and atopy. Significant values are shown in bold.

**P-values after multiple testing corrections (Meff = 42.7377).

MAF, minor allele frequency; OR, odds ratio; CI, confidence interval; NS, not significant.

## Discussion

The current study shows for the first time that *EMID2 *may be associated with the pathogenesis of nasal polyps in the onset of asthma. *EMID2_BL1_ht2*, comprising alleles of nominally significant SNPs, *rs6945102*, *rs4729697*, *rs221*, and *rs10435333*, was found to be significantly associated with nasal polyposis in the overall Korean asthma patients even after multiple testing corrections, suggesting that the variant may be a marker for inflammation of the nasal mucosa and paranasal sinuses.

With the prevalence of nasal polyps in aspirin-hypersensitive asthma patients [2], development of polyps in AERD and ATA subgroups were investigated further. Although the association signals detected in the analyses of both AERD and ATA subgroups were not deemed significant after multiple comparisons, four *EMID2 *variations (*rs6949799*, *rs4727494*, *rs13233066*, and *EMID2_BL2_ht2*) showing nominal association signals with polyp development in the AERD group in the current study was also marginally implicated in the risk of AERD in a Korean population using various modes of genetic inheritance [3], except for *EMID2_BL2_ht2 *which remained significant after multiple testing corrections in the previous report. These findings suggest that the possible role of *EMID2 *in the onset of AERD may be related to its function in the occurrence of nasal polyps in aspirin-induced asthmatics. However, due to the lack of more potent association signals reported in the current and previous studies and since the genetic make-up of individuals varies according to geographical and racial factors, the relationship between *EMID2 *and diseases of the upper and lower airways needs to be reevaluated in independent cohorts with larger sample sizes.

Recently identified as a member of the collagen protein family, EMID2 possesses Gly-*X*-Arg triplets in the collagen triple helix allowing for interaction with the heat shock protein 47 (HSP47) [5], a molecular chaperone that functions in collagen biosynthesis. Although the involvement of *EMID2 *in various human diseases remains to be elucidated, the relevance of ECM deposition and subepithelial fibrosis in diseases of the airways as well as the expression of HSP47 in nasal mucosa [6] suggest that *EMID2 *may play a role in the development of nasal polyps. In an attempt to functionally characterize the polymorphisms analyzed in the current study, *in silico *analysis was performed using the European Molecular Biology Laboratory-European Bioinformatics Institute (EMBL-EBI) splice site prediction (http://www.ebi.ac.uk/asd-srv/wb.cgi?method = 2). Refuting the common concept that intronic SNPs have no role in protein function, these intronic variants have been reported to induce alternative splicing or affect splicing efficiency [13,14]. Among the *EMID2 *SNPs showing association signals, the TTG[G >**A**]T sequence containing the 'A' allele of *rs4727494G > A *in intron 2 was observed to be a potential branch point (BP) site for alternative splicing with a BP score of 6.15, suggesting that the polymorphism may affect protein synthesis through cis-regulated alternative splicing processes. This allele is unique to *EMID2_BL2_ht2*, a haplotype that was also marginally correlated with nasal polyposis in the overall asthma patients in the current study and was previously implicated in AERD pathogenesis [3]. On the other hand, the highest scoring SNPs (*rs6945102C > T *and *rs4729697A > G*) that are unique to *EMID2_BL1_ht2*, the only *EMID2 *variant that remained significant after multiple testing corrections, were not predicted to be BP sites for alternative splicing.

After performing power calculations of single associations, the average statistical power to detect the effect sizes of the significantly associated SNPs was 69.59% (Table 2 and Table S2), suggesting insufficient sample size. Thus, the possibility of obtaining false negative findings cannot be excluded. However, in order to address this limitation and to analyze the effect of the polymorphisms in other ethnic groups, further replications in larger sample scales are required.

## Conclusions

Although the results failed to provide convincing association signals from *EMID2 *polymorphisms, the current findings report that *EMID2_BL1_ht2 *is a susceptibility marker of nasal polyposis in Korean asthma patients. The conclusions derived from the study are preliminary and may provide useful insights on the pathogenesis of nasal polyps.

## Competing interests

The authors declare that they have no competing interests.

## Authors' contributions

CFP and JSB developed tables/figures, carried out data interpretation, provided rationale for the study, and drafted the manuscript. BLP and HSC performed the statistical analysis. ASJ and STU collected the data. JHK assisted in data interpretation and drafting of the manuscript. CSP and HDS conceived the study, participated in its design, and helped to draft the manuscript. All authors read and approved the final manuscript.

## Pre-publication history

The pre-publication history for this paper can be accessed here:

http://www.biomedcentral.com/1471-2350/13/2/prepub

## Supplementary Material

Additional file 1 **Table S1 Genotype distribution of *EMID2 *polymorphisms**. Click here for file

Additional file 2 **Figure S1**. Haplotypes of *EMID2*. Haplotypes of 49 SNPs in the *EMID2 *gene obtained from four haplotype blocks. This data has been presented in our previous publication [3]. Click here for file

## References

[B1] Kakoi H Hiraide F A histological study of formation and growth of nasal polyps Acta Otolaryngol 1987 103 1-2 137 144 10.3109/00016488709134709 3551481

[B2] Hedman J Kaprio J Poussa T Nieminen MM Prevalence of asthma, aspirin intolerance, nasal polyposis and chronic obstructive pulmonary disease in a population-based study Int J Epidemiol 1999 28 4 717 722 10.1093/ije/28.4.717 10480701

[B3] Pasaje CF Kim JH Park BL Cheong HS Kim MK Choi IS Cho SH Hong CS Lee YW Lee JY A possible association of EMID2 polymorphisms with aspirin hypersensitivity in asthma Immunogenetics 2010 63 1 13 21 21086123 10.1007/s00251-010-0490-8

[B4] Braghetta P Ferrari A De Gemmis P Zanetti M Volpin D Bonaldo P Bressan GM Overlapping, complementary and site-specific expression pattern of genes of the EMILIN/Multimerin family Matrix Biol 2004 22 7 549 556 10.1016/j.matbio.2003.10.005 14996434

[B5] Sato K Yomogida K Wada T Yorihuzi T Nishimune Y Hosokawa N Nagata K Type XXVI collagen, a new member of the collagen family, is specifically expressed in the testis and ovary J Biol Chem 2002 277 40 37678 37684 10.1074/jbc.M205347200 12145293

[B6] Smirnov G Pirinen R Tuomilehto H Seppa J Terasvirta M Uusitalo H Nuutinen J Kaarniranta K Strong expression of HSP47 in metaplastic nasal mucosa may predict a poor outcome after primary endoscopic dacryocystorhinostomy: a prospective study Acta Ophthalmol 2011 89 2 e132 136 10.1111/j.1755-3768.2009.01654.x 19785638

[B7] Pasaje CF Bae JS Park BL Jang AS Uh ST Kim MK Koh IS Kim JH Park TJ Lee JS Association analysis of DTD1 gene variations with aspirin-intolerance in asthmatics Int J Mol Med 2011 28 1 129 137 21479357 10.3892/ijmm.2011.669

[B8] Livak KJ Allelic discrimination using fluorogenic probes and the 5' nuclease assay Genet Anal 1999 14 5-6 143 149 10.1016/S1050-3862(98)00019-9 10084106

[B9] Stephens M Smith NJ Donnelly P A new statistical method for haplotype reconstruction from population data Am J Hum Genet 2001 68 4 978 989 10.1086/319501 11254454 PMC1275651

[B10] Barrett JC Fry B Maller J Daly MJ Haploview: analysis and visualization of LD and haplotype maps Bioinformatics 2005 21 2 263 265 10.1093/bioinformatics/bth457 15297300

[B11] Menashe I Rosenberg PS Chen BE PGA: power calculator for case-control genetic association analyses BMC Genet 2008 9 36 10.1186/1471-2156-9-36 PMC2387159 18477402

[B12] Nyholt DR A simple correction for multiple testing for single-nucleotide polymorphisms in linkage disequilibrium with each other Am J Hum Genet 2004 74 4 765 769 10.1086/383251 14997420 PMC1181954

[B13] Maquat LE The power of point mutations Nat Genet 2001 27 1 5 6 10.1038/83759 11137984

[B14] Pagani F Baralle FE Genomic variants in exons and introns: identifying the splicing spoilers Nat Rev Genet 2004 5 5 389 396 10.1038/nrg1327 15168696

