# Supplementary material for: Possible role of EMID2 on nasal polyps pathogenesis in Korean asthma patients
Source: BMC Med Genet. 2012 Jan 4;13:2. doi: 10.1186/1471-2350-13-2 (PMC3398310; doi:10.1186/1471-2350-13-2)
Supplement: Additional file 1 — Table S1 Genotype distribution of EMID2 polymorphisms. [file 1471-2350-13-2-S1.DOCX]

Table S1 Genotype distribution of *EMID2* polymorphisms

| SNP | Position | Genotype | | | | Heterozygosity |
| --- | --- | --- | --- | --- | --- | --- |
| *rs6945102* | Intron1 | CC (238) | CT (187) | TT (42) | N (467) | 0.412 |
| *rs4729697* | Intron1 | AA (238) | AG (184) | GG (42) | N (464) | 0.411 |
| *rs10237610* | Intron1 | CC (130) | CT (228) | TT (108) | N (466) | 0.499 |
| *rs9986717* | Intron1 | CC (384) | AC (80) | AA (0) | N (464) | 0.158 |
| *rs10254516* | Intron1 | TT (384) | CT (80) | CC (3) | N (467) | 0.167 |
| *rs10239458* | Intron1 | GG (384) | AG (80) | AA (3) | N (467) | 0.167 |
| *rs221* | Intron1 | GG (226) | AG (193) | AA (47) | N (466) | 0.426 |
| *rs10435333* | Intron1 | GG (226) | AG (193) | AA (47) | N (466) | 0.426 |
| *rs6944691* | Intron1 | AA (282) | AG (168) | GG (17) | N (467) | 0.339 |
| *rs6942770* | Intron1 | TT (355) | AT (108) | AA (4) | N (467) | 0.218 |
| *rs9640666* | Intron1 | GG (173) | AG (214) | AA (80) | N (467) | 0.480 |
| *rs6947185* | Intron1 | AA (119) | AC (230) | CC (117) | N (466) | 0.500 |
| *rs11770876* | Intron1 | GG (361) | GT (103) | TT (3) | N (467) | 0.206 |
| *rs11772022* | Intron1 | GG (366) | AG (94) | AA (6) | N (466) | 0.202 |
| *rs11772003* | Intron1 | CC (367) | CG (92) | GG (6) | N (465) | 0.199 |
| *rs10223928* | Intron1 | CC (423) | CT (44) | TT (0) | N (467) | 0.090 |
| *rs4729705* | Intron1 | CC (140) | CT (226) | TT (101) | N (467) | 0.497 |
| *rs10254310* | Intron1 | AA (122) | AG (231) | GG (114) | N (467) | 0.500 |
| *rs4045* | Intron2 | GG (150) | AG (223) | AA (93) | N (466) | 0.493 |
| *rs6949799* | Intron2 | TT (149) | CT (224) | CC (93) | N (466) | 0.493 |
| *rs4727491* | Intron2 | GG (153) | AG (222) | AA (91) | N (466) | 0.491 |
| *rs13238748* | Intron2 | GG (140) | CG (226) | CC (101) | N (467) | 0.497 |
| *rs4727494* | Intron2 | GG (210) | AG (209) | AA (48) | N (467) | 0.440 |
| *rs13233066* | Intron2 | GG (209) | AG (205) | AA (53) | N (467) | 0.444 |
| *rs869127* | Intron3 | CC (424) | CT (41) | TT (2) | N (467) | 0.092 |
| *rs1476652* | Intron3 | AA (395) | AG (68) | GG (4) | N (467) | 0.149 |
| *rs6973489* | Intron3 | TT (216) | CT (194) | CC (56) | N (466) | 0.441 |
| *rs7802156* | Intron3 | AA (240) | AG (188) | GG (39) | N (467) | 0.407 |
| *rs10953342* | Intron3 | TT (243) | CT (182) | CC (42) | N (467) | 0.407 |
| *rs12668018* | Intron3 | CC (271) | CT (168) | TT (28) | N (467) | 0.365 |
| *rs1008064* | Intron3 | TT (150) | CT (211) | CC (105) | N (466) | 0.495 |
| *rs13232646* | Intron3 | TT (313) | CT (137) | CC (17) | N (467) | 0.299 |
| *rs1543883* | Intron3 | TT (136) | CT (223) | CC (107) | N (466) | 0.498 |
| *rs1859625* | Intron3 | CC (312) | CG (138) | GG (17) | N (467) | 0.300 |
| *rs6947089* | Intron3 | CC (444) | CT (22) | TT (1) | N (467) | 0.050 |
| *rs9969331* | Intron3 | GG (364) | AG (93) | AA (10) | N (467) | 0.213 |
| *rs12538381* | Intron3 | CC (384) | CT (77) | TT (6) | N (467) | 0.172 |
| *rs17135512* | Intron3 | AA (384) | AG (77) | GG (6) | N (467) | 0.172 |
| *rs1558015* | Intron3 | TT (308) | CT (141) | CC (18) | N (467) | 0.307 |
| *rs10250055* | Intron3 | AA (382) | AC (83) | CC (2) | N (467) | 0.169 |
| *rs6947735* | Intron3 | GG (325) | AG (130) | AA (12) | N (467) | 0.275 |
| *rs2158739* | Intron3 | CC (324) | CT (131) | TT (12) | N (467) | 0.277 |
| *rs10279545* | Intron3 | TT (367) | CT (95) | CC (5) | N (467) | 0.200 |
| *rs6945961* | Intron3 | GG (404) | AG (60) | AA (0) | N (464) | 0.121 |
| *rs13245946* | Intron3 | CC (394) | CT (67) | TT (6) | N (467) | 0.155 |
| *rs17470799* | Intron3 | GG(393) | AG(68) | AA(4) | N (465) | 0.150 |
| *rs10237510* | Intron3 | TT (371) | CT (90) | CC (5) | N (466) | 0.192 |
| *rs17135617* | Intron8 | AA (420) | AG (47) | GG (0) | N (467) | 0.096 |
| *rs17135621* | Intron9 | AA (418) | AG (48) | GG (1) | N (467) | 0.101 |

Table S2 Association of *EMID2* SNPs and haplotypes with nasal polyps in the overall asthmatic patients (n = 467)

| SNP/Haplotype | MAF | | Co-dominant | |  | Dominant | |  | Recessive | |  |
| --- | --- | --- | --- | --- | --- | --- | --- | --- | --- | --- | --- |
|  | Polyp-positive (n=158) | Polyp-negative (n=309) | OR(95%CI) | *P** | *P^corr***^* | OR(95%CI) | *P** | *P^corr***^* | OR(95%CI) | *P** | *P^corr***^* |
| *rs6945102C>T* | 0.228 | 0.322 | 0.62(0.45-0.87) | **0.005** | - | 0.60(0.40-0.91) | **0.02** | - | 0.39(0.16-0.92) | **0.03** | - |
| *rs4729697A>G* | 0.224 | 0.321 | 0.61(0.44-0.86) | **0.004** | - | 0.59(0.39-0.89) | **0.01** | - | 0.39(0.16-0.93) | **0.03** | - |
| *rs10237610C>T* | 0.525 | 0.451 | 1.30(0.98-1.73) | 0.07 | - | 1.53(0.96-2.46) | 0.07 | - | 1.32(0.83-2.11) | 0.24 | - |
| *rs9986717C>A* | 0.096 | 0.081 | 1.14(0.67-1.94) | 0.62 | - | 1.14(0.67-1.94) | 0.62 | - | - | - | - |
| *rs10254516T>C* | 0.101 | 0.087 | 1.17(0.72-1.92) | 0.53 | - | 1.17(0.69-1.96) | 0.57 | - | 1.75(0.15-19.97) | 0.65 | - |
| *rs10239458G>A* | 0.101 | 0.087 | 1.17(0.72-1.92) | 0.53 | - | 1.17(0.69-1.96) | 0.57 | - | 1.75(0.15-19.97) | 0.65 | - |
| *rs221G>A* | 0.252 | 0.337 | 0.67(0.49-0.93) | **0.02** | - | 0.64(0.43-0.97) | **0.04** | - | 0.48(0.22-1.06) | 0.07 | - |
| *rs10435333G>A* | 0.252 | 0.337 | 0.67(0.48-0.93) | **0.02** | - | 0.64(0.43-0.97) | **0.03** | - | 0.49(0.22-1.07) | 0.07 | - |
| *rs6944691A>G* | 0.225 | 0.212 | 1.11(0.78-1.59) | 0.56 | - | 1.12(0.74-1.68) | 0.60 | - | 1.21(0.42-3.52) | 0.72 | - |
| *rs6942770T>A* | 0.123 | 0.125 | 1.04(0.67-1.62) | 0.87 | - | 1.01(0.63-1.62) | 0.97 | - | 1.90(0.23-15.44) | 0.55 | - |
| *rs9640666G>A* | 0.354 | 0.424 | 0.76(0.56-1.01) | 0.06 | - | 0.71(0.47-1.08) | 0.11 | - | 0.65(0.37-1.15) | 0.14 | - |
| *rs6947185C>A* | 0.433 | 0.537 | 0.69(0.52-0.92) | **0.01** | - | 0.69(0.44-1.09) | 0.11 | - | 0.53(0.32-0.87) | **0.01** | - |
| *rs11770876G>T* | 0.120 | 0.115 | 1.14(0.72-1.80) | 0.58 | - | 1.17(0.72-1.88) | 0.53 | - | 0.73(0.05-9.91) | 0.81 | - |
| *rs11772022G>A* | 0.123 | 0.109 | 1.18(0.76-1.83) | 0.47 | - | 1.27(0.79-2.06) | 0.33 | - | 0.43(0.05-4.09) | 0.47 | - |
| *rs11772003C>G* | 0.118 | 0.109 | 1.13(0.73-1.77) | 0.58 | - | 1.22(0.75-1.99) | 0.42 | - | 0.43(0.05-4.05) | 0.46 | - |
| *rs10223928C>T* | 0.051 | 0.045 | 1.18(0.60-2.32) | 0.63 | - | 1.18(0.60-2.32) | 0.63 | - | - | - | - |
| *rs4729705C>T* | 0.411 | 0.482 | 0.77(0.58-1.03) | 0.08 | - | 0.75(0.49-1.16) | 0.19 | - | 0.65(0.39-1.09) | 0.10 | - |
| *rs10254310G>A* | 0.468 | 0.529 | 0.81(0.61-1.08) | 0.15 | - | 0.86(0.54-1.37) | 0.54 | - | 0.65(0.40-1.05) | 0.08 | - |
| *rs4045G>A* | 0.484 | 0.416 | 1.24(0.94-1.65) | 0.14 | - | 1.43(0.92-2.23) | 0.11 | - | 1.23(0.75-2.01) | 0.42 | - |
| *rs6949799T>C* | 0.487 | 0.416 | 1.25(0.94-1.66) | 0.12 | - | 1.45(0.93-2.26) | 0.10 | - | 1.24(0.76-2.03) | 0.39 | - |
| *rs4727491G>A* | 0.481 | 0.409 | 1.25(0.94-1.66) | 0.13 | - | 1.44(0.93-2.24) | 0.11 | - | 1.23(0.75-2.03) | 0.41 | - |
| *rs13238748G>C* | 0.411 | 0.482 | 0.77(0.58-1.03) | 0.08 | - | 0.75(0.49-1.16) | 0.19 | - | 0.65(0.39-1.09) | 0.10 | - |
| *rs4727494G>A* | 0.263 | 0.359 | 0.65(0.47-0.90) | **0.009** | - | 0.65(0.43-0.98) | **0.04** | - | 0.40(0.18-0.87) | **0.02** | - |
| *rs13233066G>A* | 0.275 | 0.362 | 0.70(0.51-0.95) | **0.02** | - | 0.65(0.43-0.97) | **0.04** | - | 0.60(0.30-1.19) | 0.14 | - |
| *rs869127C>T* | 0.044 | 0.050 | 0.98(0.51-1.89) | 0.95 | - | 0.96(0.47-1.94) | 0.90 | - | 1.44(0.08-26.23) | 0.81 | - |
| *rs1476652A>G* | 0.082 | 0.081 | 1.04(0.62-1.72) | 0.89 | - | 0.91(0.52-1.60) | 0.74 | - | 6.99(0.69-71.28) | 0.10 | - |
| *rs6973489T>C* | 0.348 | 0.318 | 1.22(0.91-1.63) | 0.19 | - | 1.18(0.78-1.77) | 0.43 | - | 1.59(0.88-2.89) | 0.13 | - |
| *rs7802156A>G* | 0.307 | 0.273 | 1.25(0.92-1.71) | 0.15 | - | 1.27(0.85-1.91) | 0.25 | - | 1.55(0.77-3.12) | 0.22 | - |
| *rs10953342T>C* | 0.297 | 0.278 | 1.16(0.85-1.58) | 0.34 | - | 1.14(0.76-1.70) | 0.54 | - | 1.46(0.74-2.90) | 0.28 | - |
| *rs12668018C>T* | 0.256 | 0.231 | 1.20(0.86-1.66) | 0.29 | - | 1.24(0.82-1.86) | 0.31 | - | 1.28(0.56-2.95) | 0.56 | - |
| *rs1008064T>C* | 0.494 | 0.430 | 1.32(1.00-1.75) | **0.05** | - | 1.46(0.94-2.26) | 0.10 | - | 1.48(0.92-2.38) | 0.10 | - |
| *rs13232646T>C* | 0.199 | 0.175 | 1.19(0.83-1.71) | 0.33 | - | 1.26(0.82-1.92) | 0.29 | - | 1.12(0.39-3.21) | 0.84 | - |
| *rs1543883T>C* | 0.506 | 0.450 | 1.32(1.00-1.76) | **0.05** | - | 1.51(0.96-2.38) | 0.08 | - | 1.41(0.88-2.25) | 0.16 | - |
| *rs1859625C>G* | 0.203 | 0.175 | 1.22(0.86-1.75) | 0.27 | - | 1.30(0.85-1.98) | 0.22 | - | 1.12(0.39-3.21) | 0.84 | - |
| *rs6947089C>T* | 0.028 | 0.024 | 1.13(0.48-2.65) | 0.78 | - | 0.98(0.39-2.47) | 0.97 | - | - | - | - |
| *rs9969331G>A* | 0.123 | 0.120 | 1.06(0.70-1.61) | 0.79 | - | 0.99(0.61-1.61) | 0.96 | - | 1.92(0.52-7.06) | 0.33 | - |
| *rs12538381C>T* | 0.095 | 0.095 | 1.04(0.65-1.66) | 0.89 | - | 0.91(0.54-1.55) | 0.73 | - | 4.22(0.71-25.08) | 0.11 | - |
| *rs17135512A>G* | 0.095 | 0.095 | 1.04(0.65-1.66) | 0.89 | - | 0.91(0.54-1.55) | 0.73 | - | 4.22(0.71-25.08) | 0.11 | - |
| *rs1558015T>C* | 0.209 | 0.180 | 1.21(0.85-1.72) | 0.30 | - | 1.24(0.81-1.88) | 0.32 | - | 1.35(0.49-3.70) | 0.56 | - |
| *rs10250055A>C* | 0.114 | 0.083 | 1.43(0.88-2.33) | 0.15 | - | 1.42(0.85-2.36) | 0.18 | - | 2.88(0.17-47.81) | 0.46 | - |
| *rs6947735G>A* | 0.177 | 0.159 | 1.16(0.79-1.69) | 0.45 | - | 1.16(0.75-1.78) | 0.51 | - | 1.45(0.43-4.86) | 0.55 | - |
| *rs2158739C>T* | 0.177 | 0.160 | 1.15(0.79-1.68) | 0.48 | - | 1.14(0.74-1.76) | 0.55 | - | 1.45(0.43-4.86) | 0.55 | - |
| *rs10279545T>C* | 0.114 | 0.112 | 1.09(0.69-1.72) | 0.71 | - | 1.00(0.61-1.63) | 0.99 | - | 4.30(0.70-26.51) | 0.12 | - |
| *rs6945961G>A* | 0.067 | 0.064 | 1.03(0.57-1.88) | 0.92 | - | 1.03(0.57-1.88) | 0.92 | - | - | - | - |
| *rs13245946C>T* | 0.108 | 0.073 | 1.53(0.95-2.45) | 0.08 | - | 1.36(0.79-2.33) | 0.26 | - | 13.01(1.45-116.41) | **0.02** | - |
| *rs17470799G>A* | 0.080 | 0.083 | 1.07(0.64-1.79) | 0.80 | - | 1.00(0.57-1.76) | 1.00 | - | 2.93(0.40-21.28) | 0.29 | - |
| *rs10237510T>C* | 0.108 | 0.107 | 1.05(0.66-1.66) | 0.85 | - | 0.95(0.57-1.57) | 0.83 | - | 4.30(0.70-26.54) | 0.12 | - |
| *rs17135617A>G* | 0.060 | 0.045 | 1.47(0.77-2.81) | 0.24 | - | 1.47(0.77-2.81) | 0.24 | - | - | - | - |
| *rs17135621A>G* | 0.063 | 0.049 | 1.35(0.73-2.51) | 0.34 | - | 1.28(0.67-2.42) | 0.46 | - | - | - | - |
| *EMID2_BL1_ht1* | 0.503 | 0.424 | 1.31(0.99-1.73) | 0.06 | - | 1.53(0.97-2.39) | 0.07 | - | 1.35(0.84-2.17) | 0.22 | - |
| *EMID2_BL1_ht2* | 0.180 | 0.290 | 0.54(0.37-0.77) | **0.0008** | 0.03 | 0.53(0.35-0.82) | **0.004** | - | 0.22(0.06-0.74) | **0.01** | - |
| *EMID2_BL1_ht3* | 0.114 | 0.112 | 1.09(0.68-1.73) | 0.72 | - | 1.11(0.68-1.80) | 0.68 | - | 0.73(0.05-9.91) | 0.81 | - |
| *EMID2_BL1_ht4* | 0.101 | 0.087 | 1.17(0.72-1.92) | 0.53 | - | 1.17(0.69-1.96) | 0.57 | - | 1.75(0.15-19.97) | 0.65 | - |
| *EMID2_BL2_ht1* | 0.475 | 0.409 | 1.22(0.92-1.62) | 0.16 | - | 1.38(0.89-2.14) | 0.15 | - | 1.23(0.74-2.02) | 0.42 | - |
| *EMID2_BL2_ht2* | 0.259 | 0.356 | 0.64(0.47-0.89) | **0.007** | - | 0.63(0.42-0.95) | **0.03** | - | 0.42(0.19-0.92) | **0.03** | - |
| *EMID2_BL2_ht3* | 0.079 | 0.061 | 1.26(0.72-2.21) | 0.42 | - | 1.21(0.67-2.17) | 0.53 | - | - | - | - |
| *EMID2_BL2_ht4* | 0.057 | 0.061 | 0.91(0.50-1.66) | 0.77 | - | 1.01(0.53-1.90) | 0.99 | - | - | - | - |
| *EMID2_BL2_ht5* | 0.044 | 0.050 | 0.98(0.51-1.89) | 0.95 | - | 0.96(0.47-1.94) | 0.90 | - | 1.44(0.08-26.23) | 0.81 | - |
| *EMID2_BL3_ht1* | 0.453 | 0.510 | 0.74(0.56-0.98) | **0.03** | - | 0.66(0.42-1.02) | 0.06 | - | 0.67(0.42-1.08) | 0.10 | - |
| *EMID2_BL3_ht2* | 0.241 | 0.212 | 1.23(0.87-1.73) | 0.24 | - | 1.28(0.85-1.93) | 0.24 | - | 1.29(0.51-3.25) | 0.59 | - |
| *EMID2_BL3_ht3* | 0.073 | 0.070 | 1.04(0.60-1.81) | 0.89 | - | 0.94(0.52-1.69) | 0.84 | - | - | - | - |
| *EMID2_BL4_ht1* | 0.237 | 0.202 | 1.26(0.90-1.77) | 0.17 | - | 1.87(0.79-4.43) | 0.15 | - | 1.24(0.82-1.87) | 0.31 | - |
| *EMID2_BL4_ht2* | 0.066 | 0.063 | 1.03(0.57-1.85) | 0.92 | - | 0.96(0.53-1.76) | 0.90 | - | - | - | - |

**P*-values at 0.05 level of significance adjusted for initial diagnosed age, sex, smoking status, atopy and AERD. Significant values are shown in bold.

***P*-values after multiple testing corrections (Meff = 42.7377).

MAF, minor allele frequency; OR, odds ratio; CI, confidence interval; NS, not significant.

Table S3 Association of *EMID2* SNPs and haplotypes with nasal polyps in AERD patients (n = 114)

| SNP/Haplotype | MAF | | Co-dominant | | | Dominant | | | Recessive | | |
| --- | --- | --- | --- | --- | --- | --- | --- | --- | --- | --- | --- |
|  | Polyp-positive (n=66) | Polyp-negative (n=48) | OR(95%CI) | *P** | *P^corr**^* | OR(95%CI) | *P** | *P^corr**^* | OR(95%CI) | *P** | *P^corr**^* |
| *rs6945102C>T* | 0.205 | 0.365 | 0.43(0.22-0.84) | **0.01** | NS | 0.39(0.17-0.86) | **0.02** | NS | 0.27(0.05-1.51) | 0.14 | - |
| *rs4729697A>G* | 0.200 | 0.365 | 0.42(0.22-0.83) | **0.01** | NS | 0.38(0.17-0.84) | **0.02** | NS | 0.27(0.05-1.57) | 0.15 | - |
| *rs10237610C>T* | 0.554 | 0.438 | 1.82(1.01-3.29) | **0.05** | NS | 1.50(0.60-3.73) | 0.38 | - | 3.42(1.22-9.60) | **0.02** | NS |
| *rs9986717C>A* | 0.114 | 0.083 | 1.29(0.47-3.53) | 0.62 | - | 1.29(0.47-3.53) | 0.62 | - | - | - | - |
| *rs10254516T>C* | 0.114 | 0.083 | 1.29(0.47-3.53) | 0.62 | - | 1.29(0.47-3.53) | 0.62 | - | - | - | - |
| *rs10239458G>A* | 0.114 | 0.083 | 1.29(0.47-3.53) | 0.62 | - | 1.29(0.47-3.53) | 0.62 | - | - | - | - |
| *rs221G>A* | 0.208 | 0.396 | 0.35(0.18-0.70) | **0.003** | NS | 0.35(0.16-0.78) | **0.01** | NS | 0.10(0.01-0.87) | **0.04** | NS |
| *rs10435333G>A* | 0.212 | 0.396 | 0.36(0.18-0.71) | **0.003** | NS | 0.36(0.16-0.81) | **0.01** | NS | 0.09(0.01-0.85) | **0.04** | NS |
| *rs6944691A>G* | 0.242 | 0.167 | 1.51(0.71-3.20) | 0.29 | - | 1.39(0.61-3.12) | 0.43 | - | - | - | - |
| *rs6942770T>A* | 0.129 | 0.083 | 1.50(0.59-3.82) | 0.40 | - | 1.46(0.55-3.84) | 0.44 | - | - | - | - |
| *rs9640666G>A* | 0.326 | 0.479 | 0.46(0.25-0.86) | **0.02** | NS | 0.35(0.15-0.84) | **0.02** | NS | 0.43(0.14-1.35) | 0.15 | - |
| *rs6947185C>A* | 0.400 | 0.531 | 0.52(0.28-0.94) | **0.03** | NS | 0.27(0.10-0.74) | **0.01** | NS | 0.65(0.25-1.69) | 0.37 | - |
| *rs11770876G>T* | 0.121 | 0.063 | 1.83(0.66-5.08) | 0.25 | - | 1.80(0.63-5.16) | 0.27 | - | - | - | - |
| *rs11772022G>A* | 0.121 | 0.104 | 1.06(0.44-2.59) | 0.90 | - | 1.25(0.48-3.24) | 0.65 | - | - | - | - |
| *rs11772003C>G* | 0.108 | 0.104 | 0.91(0.37-2.26) | 0.84 | - | 1.06(0.40-2.81) | 0.91 | - | - | - | - |
| *rs10223928C>T* | 0.053 | 0.031 | 2.00(0.46-8.61) | 0.35 | - | 2.00(0.46-8.61) | 0.35 | - | - | - | - |
| *rs4729705C>T* | 0.371 | 0.521 | 0.50(0.28-0.88) | **0.02** | NS | 0.25(0.10-0.63) | **0.003** | NS | 0.66(0.25-1.70) | 0.38 | - |
| *rs10254310G>A* | 0.432 | 0.552 | 0.58(0.34-1.01) | 0.06 | - | 0.39(0.15-0.98) | **0.04** | NS | 0.60(0.25-1.46) | 0.26 | - |
| *rs4045G>A* | 0.538 | 0.417 | 1.70(0.98-2.93) | 0.06 | - | 1.69(0.72-3.98) | 0.23 | - | 2.54(0.98-6.61) | 0.06 | - |
| *rs6949799T>C* | 0.546 | 0.417 | 1.77(1.02-3.08) | **0.04** | NS | 1.83(0.77-4.34) | 0.17 | - | 2.61(1.00-6.80) | **0.05** | NS |
| *rs4727491G>A* | 0.538 | 0.417 | 1.74(1.00-3.05) | **0.05** | NS | 1.83(0.77-4.34) | 0.17 | - | 2.48(0.95-6.51) | 0.06 | - |
| *rs13238748G>C* | 0.371 | 0.521 | 0.50(0.28-0.88) | **0.02** | NS | 0.25(0.10-0.63) | **0.003** | NS | 0.66(0.25-1.70) | 0.38 | - |
| *rs4727494G>A* | 0.220 | 0.385 | 0.46(0.25-0.86) | **0.01** | NS | 0.37(0.16-0.84) | **0.02** | NS | 0.36(0.10-1.37) | 0.14 | - |
| *rs13233066G>A* | 0.227 | 0.385 | 0.49(0.27-0.90) | **0.02** | NS | 0.37(0.16-0.84) | **0.02** | NS | 0.45(0.13-1.58) | 0.21 | - |
| *rs869127C>T* | 0.030 | 0.031 | 1.03(0.26-4.08) | 0.96 | - | 1.52(0.26-8.96) | 0.64 | - | - | - | - |
| *rs1476652A>G* | 0.076 | 0.083 | 1.01(0.38-2.71) | 0.98 | - | 0.86(0.30-2.52) | 0.79 | - | - | - | - |
| *rs6973489T>C* | 0.333 | 0.250 | 1.47(0.83-2.58) | 0.19 | - | 1.58(0.73-3.42) | 0.25 | - | 1.94(0.56-6.79) | 0.30 | - |
| *rs7802156A>G* | 0.295 | 0.219 | 1.45(0.80-2.64) | 0.22 | - | 1.82(0.83-4.00) | 0.13 | - | 1.19(0.32-4.48) | 0.80 | - |
| *rs10953342T>C* | 0.295 | 0.229 | 1.45(0.79-2.67) | 0.23 | - | 1.63(0.75-3.57) | 0.22 | - | 1.55(0.37-6.52) | 0.55 | - |
| *rs12668018C>T* | 0.265 | 0.198 | 1.48(0.78-2.81) | 0.23 | - | 1.86(0.84-4.14) | 0.13 | - | 1.01(0.22-4.58) | 0.99 | - |
| *rs1008064T>C* | 0.477 | 0.396 | 1.43(0.83-2.47) | 0.20 | - | 1.67(0.73-3.81) | 0.22 | - | 1.54(0.58-4.10) | 0.38 | - |
| *rs13232646T>C* | 0.174 | 0.188 | 0.94(0.47-1.87) | 0.85 | - | 0.92(0.41-2.06) | 0.83 | - | 0.97(0.12-7.74) | 0.98 | - |
| *rs1543883T>C* | 0.477 | 0.406 | 1.38(0.80-2.37) | 0.25 | - | 1.68(0.74-3.84) | 0.22 | - | 1.36(0.52-3.55) | 0.52 | - |
| *rs1859625C>G* | 0.174 | 0.188 | 0.94(0.47-1.87) | 0.85 | - | 0.92(0.41-2.06) | 0.83 | - | 0.97(0.12-7.74) | 0.98 | - |
| *rs6947089C>T* | 0.030 | 0.021 | 1.43(0.24-8.52) | 0.70 | - | 1.43(0.24-8.52) | 0.70 | - | - | - | - |
| *rs9969331G>A* | 0.106 | 0.125 | 0.92(0.42-2.05) | 0.84 | - | 1.02(0.39-2.66) | 0.97 | - | 0.42(0.04-5.11) | 0.50 | - |
| *rs12538381C>T* | 0.076 | 0.104 | 0.82(0.34-2.02) | 0.67 | - | 0.75(0.27-2.12) | 0.59 | - | 1.19(0.07-20.54) | 0.90 | - |
| *rs17135512A>G* | 0.076 | 0.104 | 0.82(0.34-2.02) | 0.67 | - | 0.75(0.27-2.12) | 0.59 | - | 1.19(0.07-20.54) | 0.90 | - |
| *rs1558015T>C* | 0.182 | 0.208 | 0.86(0.43-1.71) | 0.67 | - | 0.82(0.37-1.82) | 0.62 | - | 0.97(0.12-7.74) | 0.98 | - |
| *rs10250055A>C* | 0.106 | 0.094 | 1.04(0.40-2.73) | 0.94 | - | 1.04(0.40-2.73) | 0.94 | - | - | - | - |
| *rs6947735G>A* | 0.174 | 0.146 | 1.22(0.58-2.56) | 0.60 | - | 1.45(0.62-3.39) | 0.40 | - | 0.42(0.04-5.11) | 0.50 | - |
| *rs2158739C>T* | 0.174 | 0.146 | 1.22(0.58-2.56) | 0.60 | - | 1.45(0.62-3.39) | 0.40 | - | 0.42(0.04-5.11) | 0.50 | - |
| *rs10279545T>C* | 0.098 | 0.083 | 1.05(0.38-2.92) | 0.92 | - | 1.05(0.38-2.92) | 0.92 | - | - | - | - |
| *rs6945961G>A* | 0.083 | 0.042 | 1.84(0.53-6.44) | 0.34 | - | 1.84(0.53-6.44) | 0.34 | - | - | - | - |
| *rs13245946C>T* | 0.083 | 0.094 | 0.97(0.38-2.51) | 0.95 | - | 0.83(0.30-2.32) | 0.72 | - | - | - | - |
| *rs17470799G>A* | 0.061 | 0.052 | 0.87(0.25-3.04) | 0.83 | - | 0.87(0.25-3.04) | 0.83 | - | - | - | - |
| *rs10237510T>C* | 0.098 | 0.083 | 0.95(0.34-2.67) | 0.92 | - | 0.95(0.34-2.67) | 0.92 | - | - | - | - |
| *rs17135617A>G* | 0.061 | 0.021 | 2.59(0.51-13.14) | 0.25 | - | 2.59(0.51-13.14) | 0.25 | - | - | - | - |
| *rs17135621A>G* | 0.061 | 0.042 | 1.21(0.33-4.43) | 0.77 | - | 1.21(0.33-4.43) | 0.77 | - | - | - | - |
| *EMID2_BL1_ht1* | 0.538 | 0.417 | 1.83(1.02-3.26) | **0.04** | NS | 1.38(0.58-3.31) | 0.47 | - | 4.23(1.42-12.57) | **0.01** | NS |
| *EMID2_BL1_ht2* | 0.159 | 0.323 | 0.37(0.18-0.77) | **0.008** | 0.03 | 0.34(0.15-0.77) | **0.01** | NS | 0.22(0.02-2.18) | 0.19 | - |
| *EMID2_BL1_ht3* | 0.121 | 0.063 | 1.83(0.66-5.08) | 0.25 | - | 1.80(0.63-5.16) | 0.27 | - | - | - | - |
| *EMID2_BL1_ht4* | 0.114 | 0.083 | 1.29(0.47-3.53) | 0.62 | - | 1.29(0.47-3.53) | 0.62 | - | - | - | - |
| *EMID2_BL2_ht1* | 0.523 | 0.417 | 1.61(0.93-2.79) | 0.09 | - | 1.57(0.68-3.67) | 0.29 | - | 2.42(0.92-6.33) | 0.07 | - |
| *EMID2_BL2_ht2* | 0.220 | 0.385 | 0.46(0.25-0.86) | **0.01** | NS | 0.37(0.16-0.84) | **0.02** | NS | 0.36(0.10-1.37) | 0.14 | - |
| *EMID2_BL2_ht3* | 0.091 | 0.063 | 1.19(0.43-3.27) | 0.74 | - | 1.02(0.32-3.23) | 0.97 | - | - | - | - |
| *EMID2_BL2_ht4* | 0.053 | 0.073 | 0.58(0.19-1.73) | 0.32 | - | 0.65(0.19-2.21) | 0.49 | - | - | - | - |
| *EMID2_BL2_ht5* | 0.030 | 0.031 | 1.03(0.26-4.08) | 0.96 | - | 1.52(0.26-8.96) | 0.64 | - | - | - | - |
| *EMID2_BL3_ht1* | 0.485 | 0.583 | 0.65(0.39-1.11) | 0.12 | - | 0.53(0.21-1.35) | 0.18 | - | 0.58(0.25-1.34) | 0.20 | - |
| *EMID2_BL3_ht2* | 0.250 | 0.177 | 1.53(0.79-2.97) | 0.20 | - | 2.04(0.90-4.60) | 0.09 | - | 0.81(0.17-3.91) | 0.79 | - |
| *EMID2_BL3_ht3* | 0.068 | 0.083 | 0.94(0.34-2.55) | 0.90 | - | 0.78(0.26-2.33) | 0.66 | - | - | - | - |
| *EMID2_BL4_ht1* | 0.197 | 0.208 | 0.88(0.45-1.71) | 0.70 | - | 0.50(0.07-3.40) | 0.48 | - | 0.94(0.42-2.10) | 0.88 | - |
| *EMID2_BL4_ht2* | 0.061 | 0.083 | 0.73(0.25-2.19) | 0.57 | - | 0.73(0.25-2.19) | 0.57 | - | - | - | - |

**P*-values at 0.05 level of significance adjusted for initial diagnosed age, sex, smoking status, and atopy. Significant values are shown in bold.

***P*-values after multiple testing corrections (Meff = 42.7377).

MAF, minor allele frequency; OR, odds ratio; CI, confidence interval; NS, not significant.

Table S4 Association of *EMID2* SNPs and haplotypes with nasal polyps in ATA patients (n = 353)

| SNP/Haplotype | MAF | | Co-dominant | | | Dominant | | | Recessive | | |
| --- | --- | --- | --- | --- | --- | --- | --- | --- | --- | --- | --- |
|  | Polyp-positive (n=92) | Polyp-negative (n=261) | OR(95%CI) | *P** | *P^corr**^* | OR(95%CI) | *P** | *P^corr**^* | OR(95%CI) | *P** | *P^corr**^* |
| *rs6945102C>T* | 0.245 | 0.314 | 0.71(0.48-1.03) | 0.07 | - | 0.72(0.44-1.16) | 0.18 | - | 0.43(0.16-1.15) | 0.09 | - |
| *rs4729697A>G* | 0.242 | 0.313 | 0.70(0.48-1.02) | 0.06 | - | 0.70(0.43-1.14) | 0.15 | - | 0.43(0.16-1.15) | 0.09 | - |
| *rs10237610C>T* | 0.505 | 0.454 | 1.23(0.88-1.73) | 0.22 | - | 1.59(0.91-2.78) | 0.10 | - | 1.09(0.62-1.93) | 0.77 | - |
| *rs9986717C>A* | 0.082 | 0.081 | 1.02(0.54-1.96) | 0.95 | - | 1.02(0.54-1.96) | 0.95 | - | - | - | - |
| *rs10254516T>C* | 0.092 | 0.088 | 1.07(0.60-1.92) | 0.82 | - | 1.05(0.56-1.98) | 0.88 | - | 1.53(0.13-17.68) | 0.73 | - |
| *rs10239458G>A* | 0.092 | 0.088 | 1.07(0.60-1.92) | 0.82 | - | 1.05(0.56-1.98) | 0.88 | - | 1.53(0.13-17.68) | 0.73 | - |
| *rs221G>A* | 0.283 | 0.326 | 0.81(0.57-1.17) | 0.27 | - | 0.81(0.50-1.30) | 0.38 | - | 0.66(0.29-1.51) | 0.33 | - |
| *rs10435333G>A* | 0.280 | 0.326 | 0.81(0.56-1.17) | 0.26 | - | 0.79(0.49-1.29) | 0.34 | - | 0.68(0.30-1.55) | 0.36 | - |
| *rs6944691A>G* | 0.212 | 0.220 | 0.95(0.63-1.45) | 0.82 | - | 0.97(0.60-1.58) | 0.91 | - | 0.78(0.21-2.88) | 0.71 | - |
| *rs6942770T>A* | 0.120 | 0.132 | 0.88(0.52-1.50) | 0.63 | - | 0.85(0.48-1.49) | 0.56 | - | 1.59(0.14-18.08) | 0.71 | - |
| *rs9640666G>A* | 0.375 | 0.414 | 0.85(0.61-1.20) | 0.36 | - | 0.86(0.52-1.40) | 0.53 | - | 0.73(0.38-1.41) | 0.35 | - |
| *rs6947185C>A* | 0.457 | 0.538 | 0.72(0.51-1.01) | 0.06 | - | 0.86(0.50-1.50) | 0.60 | - | 0.46(0.25-0.84) | **0.01** | NS |
| *rs11770876G>T* | 0.120 | 0.125 | 0.95(0.55-1.63) | 0.85 | - | 0.98(0.56-1.72) | 0.95 | - | - | 0.99 | - |
| *rs11772022G>A* | 0.125 | 0.110 | 1.17(0.70-1.96) | 0.55 | - | 1.24(0.70-2.18) | 0.47 | - | 0.76(0.08-7.07) | 0.81 | - |
| *rs11772003C>G* | 0.125 | 0.110 | 1.17(0.70-1.96) | 0.55 | - | 1.24(0.70-2.18) | 0.47 | - | 0.76(0.08-7.07) | 0.81 | - |
| *rs10223928C>T* | 0.049 | 0.048 | 1.05(0.47-2.36) | 0.90 | - | 1.05(0.47-2.36) | 0.90 | - | - | - | - |
| *rs4729705C>T* | 0.440 | 0.475 | 0.87(0.62-1.22) | 0.41 | - | 1.05(0.62-1.80) | 0.85 | - | 0.60(0.32-1.12) | 0.11 | - |
| *rs10254310G>A* | 0.495 | 0.525 | 0.89(0.63-1.25) | 0.49 | - | 1.14(0.64-2.04) | 0.65 | - | 0.64(0.36-1.13) | 0.12 | - |
| *rs4045G>A* | 0.446 | 0.415 | 1.13(0.81-1.59) | 0.48 | - | 1.40(0.83-2.36) | 0.21 | - | 0.92(0.49-1.72) | 0.79 | - |
| *rs6949799T>C* | 0.446 | 0.416 | 1.13(0.80-1.59) | 0.48 | - | 1.40(0.83-2.35) | 0.21 | - | 0.92(0.49-1.72) | 0.80 | - |
| *rs4727491G>A* | 0.440 | 0.408 | 1.14(0.81-1.60) | 0.45 | - | 1.39(0.83-2.33) | 0.21 | - | 0.95(0.51-1.78) | 0.87 | - |
| *rs13238748G>C* | 0.440 | 0.475 | 0.87(0.62-1.22) | 0.41 | - | 1.05(0.62-1.80) | 0.85 | - | 0.60(0.32-1.12) | 0.11 | - |
| *rs4727494G>A* | 0.293 | 0.354 | 0.75(0.51-1.09) | 0.13 | - | 0.81(0.50-1.31) | 0.39 | - | 0.40(0.15-1.07) | 0.07 | - |
| *rs13233066G>A* | 0.310 | 0.358 | 0.80(0.56-1.16) | 0.24 | - | 0.80(0.49-1.29) | 0.35 | - | 0.65(0.29-1.47) | 0.30 | - |
| *rs869127C>T* | 0.054 | 0.054 | 1.00(0.47-2.12) | 0.99 | - | 0.90(0.40-1.99) | 0.79 | - | - | 0.99 | - |
| *rs1476652A>G* | 0.087 | 0.080 | 1.10(0.60-1.99) | 0.76 | - | 0.98(0.50-1.89) | 0.94 | - | 5.71(0.50-64.64) | 0.16 | - |
| *rs6973489T>C* | 0.359 | 0.331 | 1.14(0.80-1.62) | 0.46 | - | 1.06(0.66-1.72) | 0.81 | - | 1.51(0.76-3.03) | 0.24 | - |
| *rs7802156A>G* | 0.315 | 0.284 | 1.18(0.81-1.71) | 0.38 | - | 1.12(0.69-1.80) | 0.65 | - | 1.66(0.73-3.75) | 0.23 | - |
| *rs10953342T>C* | 0.299 | 0.287 | 1.08(0.75-1.55) | 0.70 | - | 1.02(0.63-1.64) | 0.95 | - | 1.37(0.62-3.04) | 0.44 | - |
| *rs12668018C>T* | 0.250 | 0.238 | 1.09(0.73-1.62) | 0.67 | - | 1.08(0.66-1.75) | 0.76 | - | 1.26(0.46-3.42) | 0.65 | - |
| *rs1008064T>C* | 0.505 | 0.437 | 1.32(0.95-1.83) | 0.10 | - | 1.43(0.84-2.43) | 0.19 | - | 1.50(0.87-2.60) | 0.15 | - |
| *rs13232646T>C* | 0.217 | 0.172 | 1.34(0.88-2.03) | 0.17 | - | 1.44(0.88-2.36) | 0.15 | - | 1.32(0.40-4.43) | 0.65 | - |
| *rs1543883T>C* | 0.527 | 0.458 | 1.34(0.96-1.88) | 0.09 | - | 1.50(0.86-2.61) | 0.16 | - | 1.46(0.85-2.53) | 0.17 | - |
| *rs1859625C>G* | 0.223 | 0.172 | 1.38(0.91-2.10) | 0.13 | - | 1.50(0.92-2.47) | 0.11 | - | 1.32(0.40-4.43) | 0.65 | - |
| *rs6947089C>T* | 0.027 | 0.025 | 1.07(0.39-2.91) | 0.90 | - | 0.86(0.27-2.72) | 0.80 | - | - | 0.99 | - |
| *rs9969331G>A* | 0.136 | 0.119 | 1.16(0.71-1.89) | 0.55 | - | 1.01(0.57-1.79) | 0.97 | - | 4.07(0.88-18.85) | 0.07 | - |
| *rs12538381C>T* | 0.109 | 0.094 | 1.18(0.69-2.05) | 0.55 | - | 1.01(0.55-1.86) | 0.99 | - | 10.96(1.08-111.20) | **0.04** | NS |
| *rs17135512A>G* | 0.109 | 0.094 | 1.18(0.69-2.05) | 0.55 | - | 1.01(0.55-1.86) | 0.99 | - | 10.96(1.08-111.20) | **0.04** | NS |
| *rs1558015T>C* | 0.228 | 0.174 | 1.40(0.93-2.12) | 0.11 | - | 1.47(0.90-2.41) | 0.12 | - | 1.69(0.55-5.22) | 0.36 | - |
| *rs10250055A>C* | 0.120 | 0.080 | 1.59(0.91-2.80) | 0.10 | - | 1.61(0.89-2.93) | 0.12 | - | 2.62(0.16-43.80) | 0.50 | - |
| *rs6947735G>A* | 0.179 | 0.161 | 1.14(0.73-1.78) | 0.57 | - | 1.06(0.63-1.76) | 0.84 | - | 2.36(0.62-9.02) | 0.21 | - |
| *rs2158739C>T* | 0.179 | 0.163 | 1.12(0.72-1.76) | 0.61 | - | 1.04(0.62-1.73) | 0.89 | - | 2.36(0.62-9.02) | 0.21 | - |
| *rs10279545T>C* | 0.125 | 0.117 | 1.07(0.64-1.79) | 0.79 | - | 0.95(0.53-1.69) | 0.86 | - | 4.12(0.67-25.42) | 0.13 | - |
| *rs6945961G>A* | 0.055 | 0.068 | 0.78(0.37-1.66) | 0.52 | - | 0.78(0.37-1.66) | 0.52 | - | - | - | - |
| *rs13245946C>T* | 0.125 | 0.069 | 1.85(1.08-3.16) | **0.02** | NS | 1.69(0.91-3.14) | 0.10 | - | 13.43(1.44-125.46) | **0.02** | NS |
| *rs17470799G>A* | 0.093 | 0.088 | 1.05(0.59-1.87) | 0.86 | - | 0.96(0.51-1.83) | 0.91 | - | 2.82(0.39-20.42) | 0.30 | - |
| *rs10237510T>C* | 0.114 | 0.112 | 1.01(0.60-1.72) | 0.96 | - | 0.88(0.48-1.59) | 0.66 | - | 4.13(0.67-25.50) | 0.13 | - |
| *rs17135617A>G* | 0.060 | 0.050 | 1.22(0.58-2.59) | 0.60 | - | 1.22(0.58-2.59) | 0.60 | - | - | - | - |
| *rs17135621A>G* | 0.065 | 0.050 | 1.33(0.66-2.72) | 0.43 | - | 1.22(0.58-2.59) | 0.60 | - | - | 0.99 | - |
| *EMID2_BL1_ht1* | 0.478 | 0.425 | 1.23(0.88-1.71) | 0.22 | - | 1.61(0.95-2.74) | 0.08 | - | 1.05(0.58-1.88) | 0.88 | - |
| *EMID2_BL1_ht2* | 0.196 | 0.284 | 0.61(0.41-0.93) | **0.02** | NS | 0.65(0.39-1.06) | 0.08 | - | 0.20(0.05-0.88) | **0.03** | NS |
| *EMID2_BL1_ht3* | 0.109 | 0.121 | 0.87(0.50-1.53) | 0.64 | - | 0.90(0.51-1.60) | 0.73 | - | . | 0.99 | - |
| *EMID2_BL1_ht4* | 0.092 | 0.088 | 1.07(0.60-1.92) | 0.82 | - | 1.05(0.56-1.98) | 0.88 | - | 1.53(0.13-17.68) | 0.73 | - |
| *EMID2_BL2_ht1* | 0.440 | 0.408 | 1.14(0.81-1.60) | 0.45 | - | 1.39(0.83-2.33) | 0.21 | - | 0.95(0.51-1.78) | 0.87 | - |
| *EMID2_BL2_ht2* | 0.288 | 0.351 | 0.74(0.50-1.08) | 0.11 | - | 0.77(0.48-1.25) | 0.30 | - | 0.43(0.16-1.16) | 0.10 | - |
| *EMID2_BL2_ht3* | 0.071 | 0.061 | 1.17(0.58-2.35) | 0.66 | - | 1.17(0.58-2.35) | 0.66 | - | - | - | - |
| *EMID2_BL2_ht4* | 0.060 | 0.059 | 1.00(0.50-2.02) | 1.00 | - | 1.08(0.51-2.27) | 0.84 | - | - | 0.99 | - |
| *EMID2_BL2_ht5* | 0.054 | 0.054 | 1.00(0.47-2.12) | 0.99 | - | 0.90(0.40-1.99) | 0.79 | - | - | 0.99 | - |
| *EMID2_BL3_ht1* | 0.429 | 0.496 | 0.76(0.54-1.06) | 0.10 | - | 0.69(0.41-1.15) | 0.15 | - | 0.69(0.38-1.24) | 0.22 | - |
| *EMID2_BL3_ht2* | 0.234 | 0.218 | 1.11(0.74-1.68) | 0.62 | - | 1.08(0.66-1.76) | 0.75 | - | 1.45(0.48-4.39) | 0.51 | - |
| *EMID2_BL3_ht3* | 0.076 | 0.067 | 1.16(0.60-2.24) | 0.65 | - | 1.07(0.54-2.13) | 0.85 | - | - | 0.99 | - |
| *EMID2_BL4_ht1* | 0.266 | 0.201 | 1.42(0.96-2.09) | 0.08 | - | 2.68(1.05-6.85) | **0.04** | NS | 1.34(0.83-2.18) | 0.24 | - |
| *EMID2_BL4_ht2* | 0.071 | 0.059 | 1.22(0.62-2.40) | 0.57 | - | 1.12(0.55-2.28) | 0.76 | - | - | 0.99 | - |

**P*-values at 0.05 level of significance adjusted for initial diagnosed age, sex, smoking status, and atopy. Significant values are shown in bold.

***P*-values after multiple testing corrections (Meff = 42.7377).

MAF, minor allele frequency; OR, odds ratio; CI, confidence interval; NS, not significant.
