# Supplementary figures and images for: Possible role of EMID2 on nasal polyps pathogenesis in Korean asthma patients
Source: BMC Med Genet. 2012 Jan 4;13:2. doi: 10.1186/1471-2350-13-2 (PMC3398310; doi:10.1186/1471-2350-13-2)

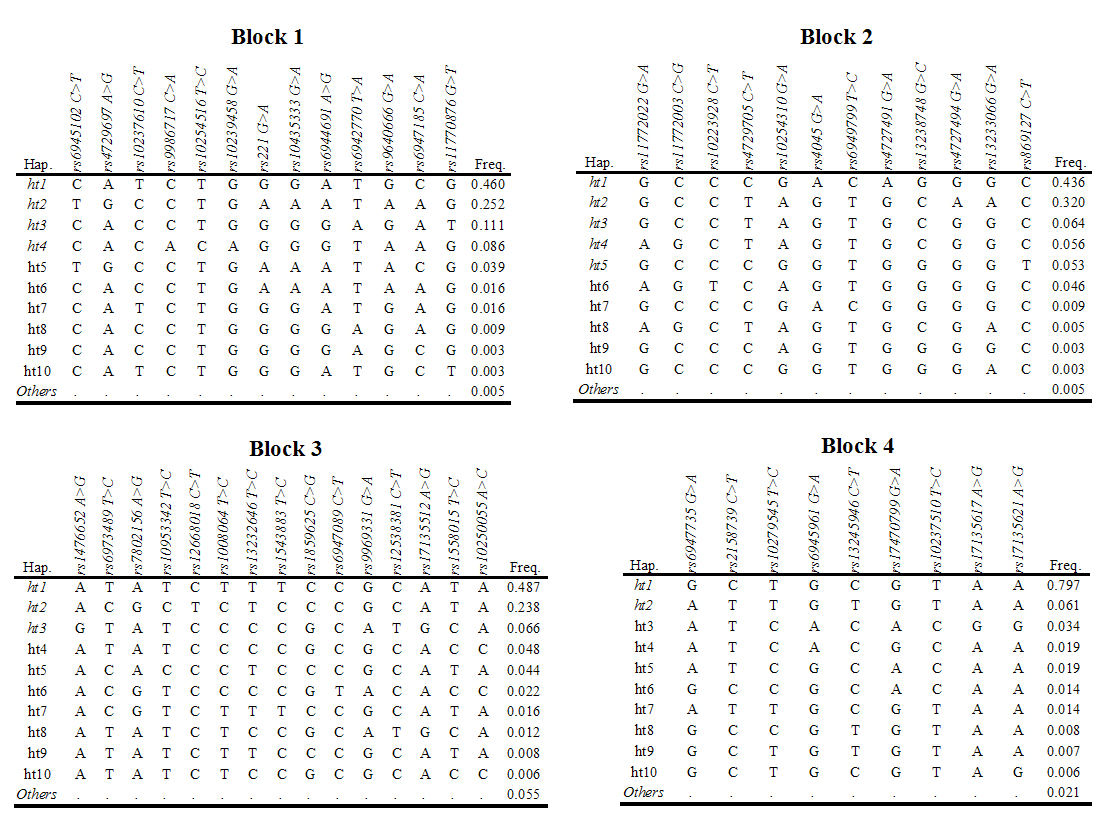

Supplement: Additional file 2 — Figure S1. Haplotypes of EMID2. Haplotypes of 49 SNPs in the EMID2 gene obtained from four haplotype blocks. This data has been presented in our previous publication [3]. [file 1471-2350-13-2-S2.JPEG]
